# Measuring Engagement with Mental Health and Behavior Change Interventions: an Integrative Review of Methods and Instruments

**DOI:** 10.1007/s12529-022-10086-6

**Published:** 2022-05-16

**Authors:** Laura Esther Bijkerk, Anke Oenema, Nicole Geschwind, Mark Spigt

**Affiliations:** 1 Department of Family Medicine, Care and Public Health Research Institute (CAPHRI), Maastricht University, Maastricht, The Netherlands; 2 Department of Health Promotion, Care and Public Health Research Institute (CAPHRI), Maastricht University, Maastricht, The Netherlands; 3 Department of Clinical Psychological Science, Maastricht University, Maastricht, The Netherlands; 4 General Practice Research Unit, Department of Community Medicine, UiT the Arctic University of Norway, Tromsø, Norway

**Keywords:** Engagement, Psychotherapy, Behavior change, Intervention, Mental health, Health behavior

## Abstract

**Background:**

Engagement is a complex construct consisting of behavioral, cognitive, and affective dimensions, making engagement a difficult construct to measure. This integrative review aims to (1) present a multidisciplinary overview of measurement methods that are currently used to measure engagement with adult mental health and behavior change interventions, delivered in-person, blended, or digitally, and (2) provide a set of recommendations and considerations for researchers wishing to study engagement.

**Methods:**

We used an integrative approach and identified original studies and reviews on engagement with mental health or behavior change interventions that were delivered in-person, digitally, or blended.

**Results:**

Forty articles were analyzed in this review. Common methods to assess engagement were through objective usage data, questionnaire-based data, and qualitative data, with objective usage data being used most frequently. Based on the synthesis of engagement measures, we advise researchers to (1) predefine the operationalization of engagement for their specific research context, (2) measure behavioral, cognitive, and affective dimensions of engagement in all cases, and (3) measure engagement over time.

**Conclusions:**

Current literature shows a bias towards behavioral measures of engagement in research, as most studies measured engagement exclusively through objective usage data, without including cognitive and affective measures of engagement. We hope that our recommendations will help to reduce this bias and to steer engagement research towards an integrated approach.

**Supplementary Information:**

The online version contains supplementary material available at 10.1007/s12529-022-10086-6.

## Introduction

Client engagement is an important and often studied process factor in intervention research. Studying client engagement is of interest because higher levels of engagement have been associated with higher levels of intervention effectiveness [1–4]. In general, engagement is described as a complex, multi-dimensional, and dynamical interaction process between a client and an intervention [5–7]. Engagement has been inconsistently defined throughout literature, but efforts have been made recently to clarify the concept of engagement [5–8]. An important next step to move the field of engagement research forward is to bring clarity and consistency in how engagement can best be measured in empirical studies.

Overall, three main dimensions of engagement can be identified: behavioral, cognitive, and affective [8]. Behavioral engagement can be characterized in terms of intervention adherence, and in terms of clients’ active effort while using the intervention [6, 7]. Adherence is described as the degree to which clients use the intervention as intended, and is usually expressed in terms of session attendance, homework compliance, or use of digital modules [9]. Effort refers to the active involvement of clients within the intervention (e.g., openly communicating relevant information and real-life behavior change) [5, 6]. Cognitive engagement refers to the degree to which clients agree with the intervention rationale, and perceive the intervention as suitable for reaching their goals [5, 10]. Affective engagement describes clients’ subjective experience with the intervention, and encompasses their experience of interest with the intervention content, the attention they pay towards the intervention, and their affective experience while interacting with the intervention [7].

Engagement is influenced by many internal and external client factors such as motivation, symptom severity, access to healthcare, and intervention characteristics [6, 11]. Other factors that influence engagement are the bond between clients and their healthcare provider [6], the social context of clients (e.g., experiencing stigma), and the intervention itself [7]. Engagement is dynamic, because levels of engagement vary throughout a therapeutic or behavior change process [5]. This means that at different stages of treatment or behavior change, different degrees of engagement and disengagement can occur. For example, some clients initially show low levels of engagement, and only start to engage more when they start to experience the results of an intervention [7]. Moreover, disengagement with an intervention does not necessarily mean disengagement with the behavior change or therapeutic process, because clients can come to a point in which they no longer need the support of the intervention [5].

To move the field of engagement research forward, it is important to identify how the various dimensions of engagement can best be measured. Engagement research is not limited to one field only. It is relevant in various domains, such as mental health [1], behavior change [12], marketing [13], education [14], human computer interaction [15], and human resources [16]. Additionally, different delivery methods for interventions have been developed in the past decades, each with different challenges and implications for engagement (e.g., digital interventions, mobile interventions, and blended interventions). The fields of mental health and behavior change differ in target population and target outcome, but are closely related, because in both cases, engagement is usually studied in an intervention context, which is not the case in the other fields mentioned. In addition, the two fields overlap, given that behavior change is an integral part of mental health recovery, and that overcoming mental obstacles is an inevitable part of behavior change.

What is missing in the literature is a multidisciplinary methodological overview of engagement in which engagement is approached as a complex, multi-dimensional, and dynamic construct. Due to its complexity, there will not be one gold-standard method to measure engagement. Instead, measurements of engagement should reflect the complexity of the construct. Therefore it is our goal to provide (1) a multidisciplinary overview of different measurement methods that are currently used to measure engagement within adult mental health and behavior change interventions, delivered in-person, blended, or digitally, and (2) a set of recommendations and considerations for researchers wishing to study engagement.

In this review, we target mental health and behavior change interventions, delivered in-person, digitally and blended. We use the term *intervention* as an umbrella term, and specify the type and delivery of the intervention for each specific study. We consider all interventions that deliver their active components through technology (e.g., smartphone, email, website, text messages, and applications) as digital interventions.

## Method

### Design

To reach the goals of this review, an integrative design was found to be best suited. An integrative review is a narrative type of literature review that allows for the critical synthesis of different literature types and perspectives, and is used to create new perspectives on a broad topic [17, 18]. A unique characteristic of integrative reviews is that they allow the researcher to combine knowledge from empirical and theoretical literature [17].

### Search Strategy

For this integrative review, we searched for studies on engagement with interventions targeting mental health conditions or behavior change in adults that were delivered in-person, digitally, or blended. Relevant literature was searched, using the following keywords referring to engagement: “Engagement,” in combination with the keywords “Intervention,” “eHealth,” “Behavior Change,” “Web-based,” “Face-to-face,” “Mental Health,” “Psychotherapy,” “CBT,” and “Blended.” The literature search was conducted between September 2019 and December 2020, with an update in February 2022, and included literature published in or after the year 2000. During this search process, several databases (EBSCO, PubMed, PsychInfo, Google Scholar) were consulted to ensure that important literature was not missed.

To be included, engagement had to be mentioned in the title or abstract of the article. We choose for this specific criterion because there are many constructs that relate to engagement, and broadening the scope would lead to an unfeasible number of articles and would lead the focus away from the main concept of interest, which is engagement. Additional literature was found using cross-referencing from the articles included in the literature search. Next to original studies, we also included literature reviews on engagement measures. Articles were excluded if the topic of interest was not on engagement with mental health or behavior change interventions, and if engagement was referred to as the engagement of clients in the healthcare decision-making process. Studies considering criminal treatment were also excluded due to the mandatory nature of these treatments.

### Literature Analysis

For analysis of the literature, we followed the methodological guidelines for integrative reviews by Whittemore and Knafl [17]. We abstracted data on research aims, target behaviors, target audience, and engagement measurement instrument from the original research. For review articles, the entire article was analyzed, and engagement measurement instruments, and their application and considerations were abstracted. The measurement instruments were then individually assessed, looking for similarities and overarching categories. We then looked for similarities and patterns within and between these categories. Lastly, all insights from the previous step were synthesized and form the basis of our practice recommendations. In an integrative review, this is part of the data analysis process and part of the “Results” section [17]. The analysis process was carried out by the first author of this review.

## Results

### How Is Engagement Measured?

This section provides an overview of the measurement methods that have been used for measuring the different dimensions of engagement, and how these methods are utilized in research practice. A total of 40 articles were included in this study, of which 37 empirical articles and 3 review articles (see Supplemental Table 1). During the literature analysis, we found that engagement measures can be generally categorized as an objective measure, a questionnaire-based measure, or a qualitative measure. The upcoming section is structured using these categories. Additionally, measurement applications for different delivery modes (i.e., in-person, digital, and blended) are provided.

#### Objective Measures

Objective measures of engagement are used to assess behavioral engagement, for example, through system usage data or client file recordings. In their simplest form, these measures reflect adherence and usage information (e.g., number of log-ins and number of sessions attended) [19]. Usage metrics with online intervention offer the possibility to collect rich usage data, with which researchers can study in-depth patterns of usage. The different options for objective adherence measures are plentiful, and depend largely on the set, setting, type of intervention, and the target population.

Adherence data for in-person interventions is often collected through recordings from an electronic client file [6]. Interventions containing a digital element typically collect adherence data through usage logs. These usage logs are automatically generated by services such as Google Analytics, or manually built into an application or website by a developer. Newer technologies allow for the recording of wearable data (i.e., Smart/sports watch and Smartphone GPS-data) in which real-time behavior can be detected and measured [19].

A useful way to categorize different objective adherence measurement options is by using the FITT (frequency, intensity, time, and type) acronym [20]. In this acronym, frequency refers to the absolute amount of contact with the intervention, for example, as the number of log-ins to an intervention. Intensity refers to the depth of contact, such as the number of exercises completed. Time refers to the amount of time spend with the intervention. Type refers to the different kinds of interaction with an intervention. For example, someone can engage in psycho-education activities by reading information or watching informational videos, which are passive activities, or someone can engage in exercises or interact with other users through social elements, which are more active activities [19]. The FITT acronym originates from eHealth and mHealth research, but is applicable to in-person interventions as well, as these interventions similarly allow for different types of engagement.

Eighteen out of the 37 studies (see Supplemental Table 1) included some sort of objective measure [21–38]. The number and type of objective measures used varied greatly from study to study. For example, Sepah et al. [24] studied the degree of engagement and the effectivity of an online diabetes prevention intervention for people diagnosed with prediabetes and measured engagement using the various adherence-related data points, such as log-ins, monitoring of relevant behaviors, and usage of the intervention’s social media functions. Others used relatively few objective measures to assess engagement in their study. For example, a study in which the association between personality characteristics and engagement with a mental health intervention was examined, measured engagement as session attendance only, and every participant that attended less than three sessions was labeled as non-engager [32]. Another study evaluated the predictive value of insomnia on drinking behavior, intervention outcomes, and intervention engagement in veterans suffering from PTSD symptoms and hazardous drinking patterns. In this study, engagement was measured as module completion only [33].

In summary, objective usage data can provide important information on adherence, a part of behavioral engagement. Objective measures are the most often used measuring methods. A benefit of objective measures is that they overcome the lack of validity and reliability of self-report measures on usage and attendance [39]. To assess effort as well as the affective and cognitive dimensions of engagement, qualitative measures and questionnaire-based measures seem to be more suitable than usage data. In the next two sections, questionnaire-based measures and qualitative measures are discussed, respectively.

#### Questionnaire-Based Measures

Questionnaire-based measures can be used to assess behavioral, cognitive, and affective dimensions of engagement. Fourteen studies made use of questionnaires to measure engagement [1, 31, 36, 40–50]. Questionnaires assessing engagement come in different forms, for example, self-report questionnaires, observer-report questionnaires, or ecological momentary assessments (EMAs) [19].

##### Self-report Questionnaires

Self-reported questionnaires are frequently used tools to assess behavioral, cognitive, and affective dimensions of engagement. Self-reported measures of adherence can be used when it is impossible to retrieve objective adherence data or to add to existing objective measures [19]. The latter option might be beneficial in case of web-based interventions in which clients may leave the intervention running in the background without actually using it. Linder et al. [43], for example, use self-report measures of adherence in their study on the impact of different digital guidance methods on engagement in an intervention targeting depression, and asked participants to estimate the total time they dedicated to the intervention each week, and how much effort they put into the intervention.

The most important pitfall of assessing adherence through self-report questionnaires is the fact that humans are not good at estimating their own intervention usage [39]. Additionally, self-report questionnaires ask about usage in retrospect, adding to the lack of reliability. When possible, objective adherence data is preferred [39]. Questionnaires also provide an option to explore clients’ effort inside and outside of the intervention, since these dimensions are harder to measure objectively.

In addition to effort, self-report questionnaires are suitable to measure cognitive and affective dimensions of engagement. Cognitive dimensions of engagement cover whether clients agree with the intervention rationale, and see the intervention as a means to reach their personal goals. Given the subjective nature of this dimension of engagement, self-report questionnaires are especially suitable. Tetley et al. [51] systematically reviewed different questionnaires assessing client engagement with face-to-face mental health interventions. Their review shows that several self-reported questionnaires measure dimensions of cognitive engagement, such as perception of progress [52, 53], intervention credibility [53], and motivation [54]. Measures also include affective dimensions such as intervention satisfaction [53], and attitude towards the intervention [52].

Also in behavior change research, there are many options to assess cognitive and affective dimensions of engagement through self-report questionnaires. For example, the DBCI Engagement Scale [46] that was developed as an instrument to measure client engagement with digital behavior change interventions asks clients to rate their levels of interest, intrigue, focus, and pleasure while using such an intervention. This scale follows the conceptualization of engagement from Perski et al. [7], in which engagement is viewed as consisting of behavioral and affective dimensions. The DBCI Engagement Scale was developed with the purpose to assess self-reported engagement with a digital health behavior change intervention, meaning that it may not be appropriate for all intervention settings [46].

Several other self-report questionnaires assess affective engagement with digital interventions [55]. Ng et al. [55] systematically reviewed measurements of engagement with mental health apps, and found several questionnaires that assessed what they called *user engagement indicators* (i.e., usability, satisfaction, acceptability, and feasibility). Two examples of the questionnaires Ng et al. [55] found that assess affective engagement are the Client Satisfaction Questionnaire (CSQ; [56]) and the System Usability Scale (SUS; [57]). Both questionnaires are short, client self-report questionnaires that assess a client’s experience with an intervention [56] or a digital product [57]. Although these questionnaires are not specifically designed to measure engagement, they do measure dimensions of engagement. Another example of a questionnaire that assesses clients’ experiences with a digital intervention is the Twente Engagement with eHealth Technologies Scale (TWEETS; [42]). The TWEETS assesses self-reported behavioral, cognitive, and affective engagement with digital interventions. The concept of affective engagement is most prominently used in the field of eHealth. This is not surprising, given that this field of research has adopted a lot of knowledge from Human Computer Interaction research, in which affective engagement is the most prominently studied concept of engagement [7].

##### Observer-Rated Questionnaires

Several examples of observer-rated questionnaires were found (see Supplemental Table 1). These questionnaires are not scored by the client, but by an observer (e.g., the therapist). This can be a useful assessment method for a construct like engagement, as the therapist might be able to give a more objective report on the fluctuating and recall-bias-prone nature of engagement. An example of an observer-rated questionnaire from the systematic review by Tetley et al. [51] is the Treatment Engagement Rating (TER; [58]). The TER is developed for forensic outpatients, but can be adapted to other contexts [58]. The TER contains nine subscales: (1) participation, (2) constructive use of sessions, (3) openness, (4) efforts to change behavior, (5) efforts to improve the socio-economic situation, (6) making sacrifices, (7) goal directedness, (8) reflection between sessions, and (9) global evaluation of treatment engagement. The engagement measure (EM; [59]) is another example of observer-rated scale that includes items that measure cognitive dimensions of engagement.

##### Ecological Momentary Assessments

Another useful application of survey data is the use of EMAs. Short et al. [19] describe the application of EMAs in their review on engagement measures for digital behavior change interventions. EMAs are repeated questionnaires with a small number of items (e.g., 3 items assessing intervention use that are sent to the client at random, prespecified, or event-contingent moments, often on a daily basis). The benefits of EMAs are that data is collected during the intervention process, instead of when the intervention period is finished, leading to reduced recall bias [19]. Another benefit of EMAs is that researchers can collect data on how engagement changes over time, thus gaining insight in the dynamic nature of engagement [5]. A pitfall of EMAs is that the repeated questionnaires can place a large burden on clients, potentially leading to missing data. EMAs are useful for assessing all subtypes of engagement. For example, clients can receive a questionnaire after every intervention contact, assessing levels of behavioral engagement, or clients can receive a questionnaire on a daily basis, assessing cognitive and affective dimensions of engagement [19].

#### Qualitative Data

Qualitative measures allow for an in-depth assessment of behavioral, cognitive, and affective dimensions of engagement. Eight studies used qualitative measures to measure engagement [38, 49, 50, 60–64]. The use of qualitative data is especially suitable for assessing out-of-session effort and the dynamic change of overall engagement over time [19]. These measures are often not directly observable by the client or the therapist, and are both considered important dimensions of engagement [5, 6]. There are different types of qualitative measures, for example, interviews, focus groups, written reviews, and think-aloud exercises. Each type has its own practicalities, benefits, and considerations.

##### Interviews

Interviews are usually carried out in a semi-structured form, meaning that a set of topics and probing questions are prepared beforehand, and the answers of the interviewee serve as points for follow-up questions. This type of qualitative data is suitable for exploring clients’ experiences of engagement and identifying ways in which the intervention can be improved, and is often used to complement quantitative data [19]. Godlaski et al. [61] explored engagement of rural women who were in the beginning stages of an outpatient substance abuse intervention, using semi-structured interviews. Several dimensions of engagement and factors relating to engagement were assessed such as participants’ attitude towards the intervention (i.e., cognitive engagement), early intervention experiences, and how those experiences influenced how comfortable the participants were with the intervention. These topics assess both the dynamic change of engagement during the intervention process [5] and the influence of the intervention’s effects on sustained engagement [7]. Instead of one interview, Gordon et al. [38] repeatedly interviewed participants who used a web-based self-help intervention for mental health and addiction complaints about their reasons for engagement and disengagement with the intervention over time. This method allowed the researchers to follow-up on participants and specifically assess the dynamic nature of engagement during the process of intervention use.

General considerations of semi-structured interviews are that they are often prone to suggestibility and recall bias, and that the process is relatively time-consuming and labor-intensive for clients and the researcher [19].

##### Focus Groups

Focus groups form another interesting option for qualitatively assessing engagement. Focus groups are small groups of participants that come together and discuss for example the engagement with an intervention. Because it is a group interview, a spontaneous discussion between participants may arise, revealing information that otherwise might have remained concealed [19]. Another benefit is that focus groups allow researchers to collect data on multiple intervention users at the same time, making it less time-consuming compared to conducting individual interviews. This type of assessment might be particularly useful for interventions that are delivered in a group setting. A limitation of this approach is that there is a high risk of social biases, such as social desirability, and polarization [19]. It might also be difficult to collect data on individual engagement using this method.

##### Other Qualitative Methods

Next to interviews and focus groups, there are several other options for qualitative research on engagement, for example, think-aloud studies, where participants are asked about their experience while using the intervention [19]. This method seems especially suitable for research on digital interventions, as there is a tactile intervention to work with (e.g., mobile app and web-based environment). Think-aloud methods are often employed in the development stages of interventions, where participant feedback is used to improve the final product [19]. Although think-aloud measures provide an important benefit, namely the reduced recall bias, there is a large risk for observer bias [19].

Other noteworthy qualitative methods are written reports and language analysis. Written reports are open-ended questions that can, for example, be added to questionnaires. This is an easy method of obtaining qualitative data, although there is no interaction possible with the participant [19]. One study by Marker et al. [63] analyzed engagement related language of clients following a transdiagnostic group intervention for anxiety disorders. Engagement related language was operationalized as language that indicated any steps towards or away from the change process (i.e., client communicates intentions to experiment with new behavior).

### Recommendations for Measuring Engagement

To conclude this section on the measurement of engagement, we provide a set of recommendations for researchers who are interested in studying engagement. First, general recommendations for engagement research are presented. In Table 1, we present measurement recommendations for behavioral engagement, cognitive engagement, and affective engagement, including their subcategories. For each type of engagement, a description of the measure, a recommendation, the setting for which the measure is appropriate, and suggestions for specific measurement tools are provided.

First, we recommend that researchers think about the specific conceptualization of engagement in the context of their study. We observed a large variety between the included studies in terms of the specific engagement measures that were used. This is not surprising, since engagement might show itself through different behaviors or attitudes, based on the intervention at study, the target population, or the applied delivery method. Additionally, engagement may be studied more extensively when treated as a primary outcome, compared to a secondary outcome. Secondly, we advise researchers to always include measures of behavioral, cognitive, and affective engagement to be able to obtain a comprehensive assessment of engagement. Measuring only behavioral, cognitive, or affective dimensions of engagement essentially means measuring adherence, intervention expectancies, or intervention experience only. Thirdly, we advise to measure engagement over time due to its dynamic nature. Engagement is a changing construct, and is theorized to be influenced by the perceived effects of an intervention [7]. Therefore, repeated measurement is important to obtain a thorough assessment of engagement.

We refrained from presenting a hierarchical recommendation in terms of specific engagement questionnaires or parameters, because studies differ in terms of setting (e.g., in-person, fully digital, and blended), form of the intervention (self-paced, individual, group), and whether engagement is studied as a primary outcome, secondary outcome, or as a mediating variable. This is also the reason why Table 1 only provides suggestions for specific measurement options. Due to the complexity of engagement as a construct, it is unlikely that a single measure can cover behavioral, cognitive, and affective engagement in an extensive and valid way. A mixed methods approach is therefore recommended for a thorough assessment of engagement.

**Table 1:** Recommendation for measuring engagement

**Measure**	**Description**	**Measurement recommendation(s)**	**Setting**	**Measurement suggestions**
**Behavioral engagement adherence**				
**Session attendance**	Number and proportion of sessions with a healthcare professional (e.g., therapist, coach, nurse, and physician) that were attended during the intervention period	Objective measured through attendance recordings of the client’s file and quantified as a proportional measure (e.g., 0.8 = 80% of sessions attended) Optionally, reasons for non-attendance can be included (e.g., no-shows indicate lower levels of engagement than cancellations within 24 h)	Interventions that include (in-person) contact appointments	1. Session attendance 2. Time in sessions 3. Therapy duration
**eHealth usage**	Usage of online intervention content, for example, by logging in, completing tasks, or emailing a therapist	Objectively measured through usage logs. Many different usage metrics are possible, depending on the intervention and the research question. It is always recommended to combine multiple usage log measures (e.g., number of log-ins + time spent in intervention)	Self-paced digital interventions, web-based guided interventions, blended interventions	1. Number of log-ins 2. Time in intervention 4. Total modules completed 5. Total number of forum posts
**Homework compliance**	The degree to which the client adheres to the tasks prescribed by the intervention/therapist	Digital interventions: usage logs of homework tasks In-person interventions: Homework diaries: client-completed diary forms in which clients record the homework tasks they have completed. The degree to which the exercise is completed is then rated in a way that is appropriate for the nature of the exercise (e.g., completed yes/no)	Interventions that include homework exercises	1. Homework specific usage metrics (e.g., number of exercises completed and number of behaviors logged) 2. Form including the date, duration, and type of homework exercise completed
**Behavioral engagement effort**				
**Discussing emotions, thoughts, and behavior (in intervention)**	Disclosure of relevant information in the intervention environment (e.g., therapist and forum)	1. (Repeated) client-rated questionnaires 2. (Repeated) interviews 3. Therapist-rated questionnaires	All types of interventions	1. Single-item questions (e.g., I openly discuss my feeling with my therapist) 2. Treatment Engagement Rating (TER; [58]) 3. Groupwork Engagement Measure (GEM; [67])
**Homework quality**	The degree to which the client has put effort into the intervention exercises	1. (Repeated) client-rated questionnaires 2. Therapist-rated questionnaires 3. (Repeated) client interviews	In-person or digital interventions	Single-item questions (e.g., I put effort in my homework exercises) Or therapist-rated questions (e.g., rate the effort your client put into the homework exercises)
**Experimenting with new behavior (outside of intervention)**	The degree to which the client actively applies the newly learned skills/techniques/insights/behaviors in daily life	1. (Repeated) interviews with clients 2. (Repeated) client-rated questionnaires 3. Wearables	All types of interventions	1. Single-item questions (e.g., I use what I’ve learned in daily life) 2. Treatment Engagement Rating (TER; [58]) 3. Pedometer on smartwatch
**Cognitive engagement identification with the intervention**				
**Identification with the intervention rationale**	The client understands how the intervention is built, what the goals of the intervention are, and how these goals are reached	1. Client-based questionnaires that are collected at the start and end of the intervention and preferably at multiple time points in-between 2. (Repeated) interviews	All types of interventions	1. Single-item questions (e.g., I understand the goals of the treatment) 2. Client Expectations Questionnaire (CEQ; [68]) 3. Client Satisfaction Questionnaire (CSQ; [56])
**Sees intervention as means to reach personal goals**	The client sees the intervention as a suitable way to reach personal treatment goals	Client-based questionnaires that are collected at the start and end of the intervention and preferably at multiple time points in-between	All types of interventions	1. Single-item questions (e.g., This intervention will help me reach my goals) 2. Client Expectations Questionnaire (CEQ; [68]) 3. Client Satisfaction Questionnaire (CSQ; [56])
**Affective engagement subjective experience with the intervention**				
**Subjective experience**	Clients’ subjective experience with the intervention in terms of their attention, interest, and affect	1. (Repeated) client questionnaires 2. (Repeated) interviews 3. Ecological Momentary Assessments	All types of interventions	1. Client Satisfaction Questionnaire (CSQ; [56]) Digital interventions: 1. User Experience Questionnaire (UEQ; [69]) 2. DBCI Engagement Scale [46]

## Discussion

The aim of this review was to provide researchers with (1) a multidisciplinary overview of different measurement methods that are currently used to measure engagement with adult mental health and behavior change interventions, delivered in-person, blended, or digitally, and (2) a set of recommendations and considerations for researchers wishing to study engagement. These recommendations form a general approach to assessment that needs to be customized for each study, taking into account aspects such as study goal, type of intervention, health condition, and client population.

The complex and multi-dimensional nature of engagement makes it a difficult construct to measure. In past research on engagement, the main focus has been on usage and adherence data. Gaining insight into how much a client uses the intervention is important when studying engagement, but covers only one part of a complex concept. Overall, we noticed a strong focus on assessing behavioral dimensions of engagement, while affective and cognitive dimensions were largely neglected, as visible in Supplemental Table 1. Holdsworth et al. [6] highlight an important restriction with using attendance as the only proxy for engagement. They state that “*it is quite plausible that clients can attend treatment, even at a level equivalent to a ‘treatment dose’ without necessarily being engaged. Equally, clients may be engaged within the therapeutic process without being present at every session, or even before they have attended the first session*” (p.431). Similar statements have been made about using usage data as a proxy for engagement in research on digital mental health interventions [65]. For example, Yardley et al. [5] introduced the concept of effective engagement in the context of digital behavior change interventions, and highlighted that engagement manifests in different ways, with intervention adherence being only one of them. Especially in later stages, clients often show engagement by enacting change in real life instead of engaging with the intervention content [5]. Although adherence is an important dimension of engagement, it is thus not suitable to be used as the only proxy for engagement. We argue that measuring engagement without including cognitive and affective dimensions is insufficient. Studies that include only objective behavioral measures are in essence measuring usage or adherence, not engagement.

Our overview of measures and recommendations comes with some practical and methodological limitations that are important to consider. A first limitation is that our recommendations mainly focus on the operationalization of engagement and not on specific measures, data points, or questionnaires. Because of this, researchers still need to find the appropriate specific measures of engagement. We did provide some examples, but refrained from a detailed list of measures. The reason for this is that the nature of engagement is highly contextual, as every intervention, population, or delivery mode requires another approach. Another reason is that this list would quickly become outdated with the fast-growing knowledge in engagement research.

For this review, we chose an integrative approach because it suited our goal to integrate knowledge from different but related disciplines. Behavior change interventions and mental health interventions share similarities in terms of their mechanisms of action and delivery methods. For example, both fields make use of eHealth to deliver the content of the intervention to the client. Also, in both fields, it is necessary for the client to actively interact with the intervention in order to get results. Due to these similarities, the expression of engagement in terms of operationalization and measures is also very similar. Therefore, we find it a logical step to integrate the knowledge from these two fields.

Additionally, we wanted to build on this integrated knowledge and shape it into the practice-oriented recommendations that we present in this paper. A limitation of an integrative review concerns the integrity of the search results. We did not conduct a fully systematic literature search, and might have missed literature. However, we want to emphasize that this work does not aim to provide an exhausting overview of measures and operationalizations, but is intended as a starting point for researchers to shape their operationalization of engagement.

Next steps in engagement research would be investigating specific predictors and facilitators of engagement, and using techniques to create interventions that are more engaging, which could ultimately improve the effectiveness of interventions. Gaining insight into engagement provides valuable information on how an intervention is used and what invites clients towards usage, thereby providing more insight into the “black box” of intervention effectiveness. Looking at process variables such as engagement also reduces the chance of a so-called type III error, in which intervention effects are erroneously missed because of low usage [66]. For a concept as broad as engagement, we feel that a multidisciplinary approach is well suited. For example, research in eHealth has taken great steps by adopting user-centered persuasive designs and using gamification as a strategy to create more engaging interventions [52, 53]. Blended interventions have become more common, combining in-person and digital components to create flexible interventions with personal support. These approaches seem very promising, but can probably be developed further. We hope that this review helps researchers in providing a starting point for making informed decisions when studying engagement, thereby facilitating developments in engagement research.

## Supplementary Information

Below is the link to the electronic supplementary material.

Supplementary file1 (DOCX 38 kb)

## Declarations

### Study with no Human or Animal Subjects

This article does not contain any studies with human participants or animals performed by any of the authors.

## References

[1] Glenn D, Golinelli D, Rose RD, Roy-Byrne P, Stein MB, Sullivan G, Bystritksy A, Sherbourne C, Craske MG (2013). Who gets the most out of cognitive behavioral therapy for anxiety disorders? The role of treatment dose and patient engagement. J Consult Clin Psychol.

[2] Hundt NE, Amspoker AB, Kraus-Schuman C, Cully JA, Rhoades H, Kunik ME, Stanley MA (2014). Predictors of CBT outcome in older adults with GAD. J Anxiety Disord.

[3] Neimeyer RA, Kazantzis N, Kassler DM, Baker KD, Fletcher R (2008). Group cognitive behavioural therapy for depression outcomes predicted by willingness to engage in homework, compliance with homework, and cognitive restructuring skill acquisition. Cogn Behav Ther.

[4] Gan DZQ, McGillivray L, Han J, Christensen H, Torok M (2021). Effect of engagement with digital interventions on mental health outcomes: a systematic review and meta-analysis. Front Digit Health..

[5] Yardley L, Spring BJ, Riper H, Morrison LG, Crane DH, Curtis K, Merchant GC, Naughton F, Blandford A (2016). Understanding and promoting effective engagement with digital behavior change interventions. Am J Prev Med.

[6] Holdsworth E, Bowen E, Brown S, Howat D (2014). Client engagement in psychotherapeutic treatment and associations with client characteristics, therapist characteristics, and treatment factors. Clin Psychol Rev.

[7] Perski O, Blandford A, West R, Michie S (2017). Conceptualising engagement with digital behaviour change interventions: a systematic review using principles from critical interpretive synthesis. Transl Behav Med.

[8] Kelders SM, van Zyl LE, Ludden GDS (2020). The concept and components of engagement in different domains applied to eHealth: a systematic scoping review. Front Psychol.

[9] World Health Organization (2003). Adherence to long-term therapies: evidence for action.

[10] Tzavela EC, Mitskidou P, Mertika A, Stalikas A, Kasvikis Y (2018). Treatment engagement in the early phase of cognitive-behavior therapy for panic disorder: a grounded theory analysis of patient experience. Psychother Res.

[11] Drieschner KH, Lammers S, van der Staak CPF (2004). Treatment motivation: an attempt for clarification of an ambiguous concept. Clin Psychol Rev.

[12] Richardson A, Graham AL, Cobb N, Xiao H, Mushro A, Abrams D, Vallone D (2013). Engagement promotes abstinence in a web-based cessation intervention: cohort study. J Med Internet Res.

[13] Hapsari R, Clemes MD, Dean D (2017). The impact of service quality, customer engagement and selected marketing constructs on airline passenger loyalty. Int J Qual Serv Sci.

[14] Tight M (2020). Student retention and engagement in higher education. J Furth High Educ.

[15] O’Brien HL, Toms EG (2008). What is user engagement? A conceptual framework for defining user engagement with technology. J Am Soc Inf Sci Technol.

[16] Anitha J (2014). Determinants of employee engagement and their impact on employee performance. Int J Product Perform Manag.

[17] Whittemore R, Knafl K (2005). The integrative review: updated methodology. J Adv Nurs.

[18] Torraco RJ (2005). Writing integrative literature reviews: guidelines and examples. Hum Resour Dev Rev.

[19] Short CE, DeSmet A, Woods C, Williams SL, Maher C, Middelweerd A, Müller AM, Wark PA, Vandelanotte C, Poppe L, Hingle MD, Crutzen R (2018). Measuring engagement in eHealth and mHealth behavior change interventions: viewpoint of methodologies. J Med Internet Res.

[20] Barisic A, Leatherdale ST, Kreiger N (2011). Importance of frequency, intensity, time and type (FITT) in physical activity assessment for epidemiological research. Can J Public Health.

[21] Arnold C, Villagonzalo K-A, Meyer D, Farhall J, Foley F, Kyrios M, Thomas N (2019). Predicting engagement with an online psychosocial intervention for psychosis: exploring individual- and intervention-level predictors. Internet Interv.

[22] Baltierra NB, Muessig KE, Pike EC, LeGrand S, Bull SS, Hightow-Weidman LB (2016). More than just tracking time: complex measures of user engagement with an internet-based health promotion intervention. J Biomed Inform.

[23] Ben-Zeev D, Scherer EA, Gottlieb JD, Rotondi AJ, Brunette MF, Achtyes ED, Mueser KT, Gingerich S, Brenner CJ, Begale M, Mohr DC, Schooler N, Marcy P, Robinson DG, Kane JM (2016). mHealth for schizophrenia: patient engagement with a mobile phone intervention following hospital discharge. JMIR Mental Health.

[24] Sepah SC, Jiang L, Ellis RJ, McDermott K, Peters AL (2017). Engagement and outcomes in a digital diabetes prevention program: 3-year update. BMJ Open Diabetes Res Care.

[25] Couper MP, Alexander GL, Zhang N, Little RJA, Maddy N, Nowak MA, McClure JB, Calvi JJ, Rolnick SJ, Stopponi MA, Johnson CC (2010). Engagement and retention: measuring breadth and depth of participant use of an online intervention. J Med Internet Res.

[26] Kouwenhoven-Pasmooij TA, Robroek SJ, Ling SW, van Rosmalen J, van Rossum EF, Burdorf A, Hunink MM (2017). A blended web-based gaming intervention on changes in physical activity for overweight and obese employees: influence and usage in an experimental pilot study. JMIR Serious Games.

[27] Matthews P, Topham P, Caleb-Solly P (2018). Interaction and engagement with an anxiety management app: analysis using large-scale behavioral data. J Med Internet Res.

[28] Murray JM, French DP, Patterson CC, Kee F, Gough A, Tang J, Hunter RF (2019). Predicting outcomes from engagement with specific components of an internet-based physical activity intervention with financial incentives: process analysis of a cluster randomized controlled trial. J Med Internet Res.

[29] Almodovar AS, Surve S, Axon DR, Cooper D, Nahata MC (2018). Self-directed engagement with a mobile app (Sinasprite) and its effects on confidence in coping skills, depression, and anxiety: retrospective longitudinal study. JMIR Mhealth Uhealth.

[30] Strecher VJ, McClure J, Alexander G, Chakraborty B, Nair V, Konkel J, Greene S, Couper M, Carlier C, Wiese C, Little R, Pomerleau C, Pomerleau O (2008). The role of engagement in a tailored web-based smoking cessation program: randomized controlled trial. J Med Internet Res.

[31] Yeager CM, Shoji K, Luszczynska A, Benight CC (2018). Engagement with a trauma recovery internet intervention explained with the health action process approach (HAPA): longitudinal study. JMIR Mental Health.

[32] Patel KD, Suhr JA (2019). The relationship of MMPI–2–RF scales to treatment engagement and alliance. J Pers Assess.

[33] Buckheit KA, Nolan J, Possemato K, Maisto S, Rosenblum A, Acosta M, Marsch LA (2022). Insomnia predicts treatment engagement and symptom change: a secondary analysis of a web-based CBT intervention for veterans with PTSD symptoms and hazardous alcohol use. Transl Behav Med..

[34] Figueroa CA, Demasi O, Hernandez-Ramos R, Aguilera A (2021). Who benefits most from adding technology to depression treatment and how? An analysis of engagement with a texting adjunct for psychotherapy. Telemed J E Health.

[35] Wols A, Hollenstein T, Lichtwarck-Aschoff A, Granic I (2021). The effect of expectations on experiences and engagement with an applied game for mental health. Games Health J.

[36] Newman MW, Hawrilenko M, Jakupcak M, Chen S, Fortney JC (2021). Access and attitudinal barriers to engagement in integrated primary care mental health treatment for rural populations. J Rural Health.

[37] Suffoletto B, Goldstein T, Gotkiewicz D, Gotkiewicz E, George B, Brent D (2021). Acceptability, engagement, and effects of a mobile digital intervention to support mental health for young adults transitioning to college: pilot randomized controlled trial. JMIR Form Res..

[38] Gordon D, Hensel J, Bouck Z, Desveaux L, Soobiah C, Saragosa M, Jeffs L, Bhatia S, Shaw J (2021). Developing an explanatory theoretical model for engagement with a web-based mental health platform: results of a mixed methods study. BMC Psychiatry.

[39] Flett JAM, Fletcher BD, Riordan BC, Patterson T, Hayne H, Conner TS (2019). The peril of self-reported adherence in digital interventions: a brief example. Internet Interv.

[40] Aizenstros A, Bakker D, Hofmann SG, Curtiss J, Kazantzis N (2021). Engagement with smartphone-delivered behavioural activation interventions: a study of the MoodMission smartphone application. Behav Cogn Psychother.

[41] Graham AK, Kwasny MJ, Lattie EG, Greene CJ, Gupta NV, Reddy M, Mohr DC (2021). Targeting subjective engagement in experimental therapeutics for digital mental health interventions. Internet Interv.

[42] Kelders SM, Kip H, Greeff J (2020). Psychometric evaluation of the Twente Engagement with Ehealth Technologies Scale (TWEETS): evaluation study. J Med Internet Res.

[43] Lindner P, Olsson EL, Johnsson A, Dahlin M, Andersson G, Carlbring P (2014). The impact of telephone versus e-mail therapist guidance on treatment outcomes, therapeutic alliance and treatment engagement in Internet-delivered CBT for depression: a randomised pilot trial. Internet Interv.

[44] Mallonee J, Petros R, Solomon P (2021). Explaining engagement in outpatient therapy among adults with serious mental health conditions by degree of therapeutic alliance, therapist empathy, and perceived coercion. Psychiatr Rehabil J.

[45] McNealy KR, Lombardero A (2019). Somatic presentation of mental health concerns, stigma, and mental health treatment engagement among college students. J Am Coll Health.

[46] Perski O, Blandford A, Garnett C, Crane D, West R, Michie S (2019). A self-report measure of engagement with digital behavior change interventions (DBCIs): development and psychometric evaluation of the “DBCI Engagement Scale”. Transl Behav Med.

[47] Saul JE, Amato MS, Cha S, Graham AL (2016). Engagement and attrition in Internet smoking cessation interventions: insights from a cross-sectional survey of “one-hit-wonders”. Internet Interv.

[48] Zelencich LM, Kazantzis N, Wong D, McKenzie DP, Downing M, Ponsford JL (2019). Predictors of homework engagement in CBT adapted for traumatic brain injury: pre/post-injury and therapy process factors. Cogn Ther Res.

[49] Lawn S, Kaine C, Stevenson J, McMahon J (2021). Australian mental health consumers’ experiences of service engagement and disengagement: a descriptive study. Int J Environ Res Public Health.

[50] Morrison L, Moss-Morris R, Michie S, Yardley L (2014). Optimizing engagement with Internet-based health behaviour change interventions: comparison of self-assessment with and without tailored feedback using a mixed methods approach. Br J Health Psychol.

[51] Tetley A, Jinks M, Huband N, Howells K (2011). A systematic review of measures of therapeutic engagement in psychosocial and psychological treatment. J Clin Psychol.

[52] Fals-Stewart W, Lam WKK (2010). Computer-assisted cognitive rehabilitation for the treatment of patients with substance use disorders: a randomized clinical trial. Exp Clin Psychopharmacol.

[53] Nelson RA, Borkovec TD (1989). Relationship of client participation to psychotherapy. J Behav Ther Exp Psychiatry.

[54] Tsang WH, Fung KMT, Corrigan PW, Tsang HWH, Fung KMT, Corrigan PW (2006). Psychosocial treatment compliance scale for people with psychotic disorders. Aust N Z J Psychiat.

[55] Ng MM, Firth J, Minen M, Torous J (2019). User engagement in mental health apps: a review of measurement, reporting, and validity. Psychiatr Serv.

[56] Attkisson CC, Zwick R (1982). The client satisfaction questionnaire: psychometric properties and correlations with service utilization and psychotherapy outcome. Eval Program Plann.

[57] Bangor A, Kortum PT, Miller JT (2008). An empirical evaluation of the system usability scale. Int J Hum Comput Interact.

[58] Drieschner KH, Boomsma A (2008). The treatment engagement rating scale (TER) for forensic outpatient treatment: description, psychometric properties, and norms. Psychol Crime Law.

[59] Hall M, Meaden A, Smith J, Jones C (2001). Brief report: the development and psychometric properties of an observer-rated measure of engagement with mental health services. J Ment Health.

[60] Fitzpatrick KK, Darcy A, Vierhile M (2017). Delivering cognitive behavior therapy to young adults with symptoms of depression and anxiety using a fully automated conversational agent (Woebot): a randomized controlled trial. JMIR Mental Health.

[61] Godlaski TM, Butler L, Heron M, Debord S, Cauvin L (2009). A qualitative exploration of engagement among rural women entering substance user treatment. Subst Use Misuse.

[62] Knowles SE, Lovell K, Bower P, Gilbody S, Littlewood E, Lester H (2015). Patient experience of computerised therapy for depression in primary care. BMJ Open.

[63] Marker I, Salvaris CA, Thompson EM, Tolliday T, Norton PJ (2019). Client motivation and engagement in transdiagnostic group cognitive behavioral therapy for anxiety disorders: predictors and outcomes. Cogn Ther Res.

[64] Soderlund PD, Hollingsworth ASM, Heilemann MSV (2021). Participant engagement in a transmedia storytelling web-based app intervention for mental health of Latina women: qualitative analysis. JMIR Mental Health..

[65] Donkin L, Hickie IB, Christensen H, Naismith SL, Neal B, Cockayne NL, Glozier N (2013). Rethinking the dose-response relationship between usage and outcome in an online intervention for depression: randomized controlled trial. J Med Internet Res.

[66] Dobson D, Cook TJ (1980). Avoiding type III error in program evaluation. Eval Program Plann.

[67] Macgowan MJ (2000). Evaluation of a measure of engagement for group work. Res Soc Work Pract.

[68] Devilly GJ, Borkovec TD (2000). Psychometric properties of the credibility/expectancy questionnaire. J Behav Ther Exp Psychiatry.

[69] Laugwitz B, Held T, Schrepp M. Construction and evaluation of a user experience questionnaire. In: Symposium of the Austrian HCI and Usability Engineering Group, Springer, 2008. pp. 63–76.

