# Supplementary material for: Measuring Engagement with Mental Health and Behavior Change Interventions: an Integrative Review of Methods and Instruments
Source: Int J Behav Med. 2022 May 16;30(2):155–66. doi: 10.1007/s12529-022-10086-6 (PMC10036274; doi:10.1007/s12529-022-10086-6)
Supplement: Supplementary file 1 — Supplementary file1 (DOCX 38 kb) [file 12529_2022_10086_MOESM1_ESM.docx]

*Supplemental Table 1. The Measurement of Engagement Across Studies.*

| **Objective Measures** | | | | | |
| --- | --- | --- | --- | --- | --- |
| Author | Research aim | Sample and intervention type | Target context | Delivery mode | Operationalization of engagement |
| (Arnold et al., 2019) | Study aimed at investigating variables that predict treatment engagement in clients following an intervention for psychosis. | 89 participants using an online intervention targeting self-help and recovery in psychosis treatment were randomized to use the intervention autonomously or with additional email support for a period of 12 weeks. | Mental health | Digital | Intervention use: Dates and times of login, activities on website  Depth of use: ﻿total amount of website use over the intervention period  Breadth: ﻿the variation in participants' use of the website  Active/passive engagement: percentage of active intervention use (e.g. posting, completing exercises) versus passive intervention use (e.g. reading a post, viewing a video) |
| (Baltierra et al., 2016) | Exploration of complementary methods to measure and assess user metrics of web-based interventions | Pilot study of a multi-component web-based intervention for young black trans man and woman that have sex with men, targeting sexual health. | Behavior change | Digital | Website utilization: time stamps of user activity (e.g. login, quiz completion)  Reputation points per user: Points were provided for active site use (e.g. submitting a question, getting a perfect score on a quiz). |
| (Ben-Zeev et al., 2016) | Examination of patient engagement with a mobile intervention supporting patients with schizophrenia after hospital discharge. | 342 participants diagnosed with schizophrenic-spectrum disorder that were assigned to use the intervention in the 6-months following hospital discharge | Mental health | Digital | ﻿1) Days of mHealth use, 2) the number of days a participant responded to system-initiated prompts during the week, 3) the number of days a participant initiated [intervention] use during the week & 4) how often within a day, individuals self-initiated [intervention] functions. |
| (Buckheit et al., 2022) | Secondary examination of the predictive value of insomnia on PTSD symptoms, alcohol use and treatment engagement with a web-based intervention targeting PTSD symptoms and hazardous drinking for veterans. | 162 veterans experiencing PTSD symptoms were randomized to receive treatment via a web-based intervention, or treatment as usual. | Mental health | Digital | Module completion |
| (Cameron Sepah et al., 2017) | Examination of intervention engagement and outcome from baseline to 3-years post baseline in an online diabetes prevention program. | 220 patients diagnosed with pre-diabetes were enrolled in a 16-week online prevention program targeting weight loss and management. | Behavior change | Digital | ﻿﻿(1) Lessons Completed; (2) the number of weeks during which a participant weighed-in at least once; (3) number of weeks during which a participant logged their daily walking steps at least once; (4) the number of comments made or replied to on the group board; (5) the number of group board comments a participant [liked]; (6) The number of unique login sessions and (7) the number of private messages sent from a participant to his or her health coach. |
| (Couper et al., 2010) | Exploration of the quality of engagement with an online intervention promoting fruit and vegetable intake | 2513 healthy participants.  Group 1: untailored web sessions  Group 2: tailored web sessions  Group 3: tailored web sessions with support | Behavior change | Digital | ﻿Time stamps for: (1) logging in to the website; (2) initiation of any of the 4 online surveys; (3) completion of any of the online surveys; (4) loading the first page of any of the core [intervention features]; and (5) loading the first page of any special features. |
| (Figueroa et al., 2021) | Study assessing different client profiles and associated engagement, response rates, and face-to-face attendance of add-on texting support in combination with group CBT. | 73 participants who followed group CBT in primary care were provided add-on texting support on top of their group CBT. Machine learning was used to detect different client profiles. | Mental health | Blended | 1) total texting responsiveness (e.g., the number of texts responded to over the whole study period) and 2) in-clinic CBT attendance. |
| (Glasgow et al., 2007) | Study assessing the enrollment rates, levels of engagement, and association between enrollment and engagement of internet-based weight loss programs. | 2x2 design in which 23 overweight members of health organizations were invited to participate in an online weight loss program through two different recruitment methods: 1) a personal letter from the prevention director, 2) a notice in the company newsletter, and then randomized to receive the intervention or not. | Behavior change | Digital | Initial engagement: participant has read the intervention instructions yes/no  Ongoing engagement: participant has read the follow-up electronic newsletter regarding the intervention yes/no |
| (Wols et al., 2021) | Study examining the effect of expectation on experiences and engagement with a game-based application for depression and anxiety. | 57 psychology students showing elevated anxiety symptoms were showed a trailer for a mental health app that was either mental health based or entertainment based. After watching the trailer, the participants played the game for 60 minutes. | Mental health | Digital | Practice of relaxation, exposure, and modifying attention biases |
| (Kouwenhoven-Pasmooij et al., 2017) | Assessment of the influence and usage of a blended game-based intervention targeting physical activity and weight loss in overweight and obese workers. | Non-randomized pre-post trial in which 52 workers with a BMI > 25 kg/m participated in a voluntary 23-week weight loss intervention, consisting of (gamified) web-based components and consults with an occupational health physician. | Behavior change | Blended | ﻿Engagement was measured as the number of times at least 100% of the personal target level was reached (ranging from 0 to 5) and categorized into ≤3 times and 4 or 5 times. |
| (Matthews et al., 2018) | Investigation of engagement and patterns of engagement of clients using a self-help app for anxiety management. | Analysis of anonymous usage data of 105,380 users of a self-help app for anxiety management | Mental health | Digital | Coding of users’ behavior in intervention: ﻿anxiety monitoring, ratings of self-help options, and [social intervention function] activity.  ﻿App users were coded into binary categories according to their engagement levels (e.g. high engager) |
| (Murray et al., 2019) | Assessment of engagement and non-usage attrition of an online workplace implemented intervention aimed at raising physical activity | 457 workers in various workplaces in Northern Ireland. | Behavior change | Digital | ﻿1) Daily physical activity captured via [intervention] physical activity monitoring system  2) Use of the [intervention] website  3) Reward redemption |
| ﻿(Patel & Suhr, 2019) | Examining the association between personality characteristics and treatment engagement/treatment alliance | Database study of 134 clients that applied for mental health treatment at a psychology student training clinic. | Mental health | In-person | 1) dichotomous categorization of terminators (clients that only attended < 3 sessions) and non-terminators (clients that attended all three sessions)  2) percentage of no show |
| (Silva Almodovar et al., 2018) | Evaluation of database data of on how treatment engagement affects coping skills and symptoms of anxiety and depression in a mobile app targeting the improvement of coping skills in depression and anxiety. | Database data of 34 users that were either referred to the intervention by a clinician or applied themselves. The intervention a self-guided, game-based mobile phone app, incorporating elements of CBT and Mindfulness. | Mental health | Digital | ﻿Several indicators were used to evaluate the mobile app.  Included were the average length of in-game session, completed  meditation sessions, mindfulness paintings, anxiety journal  entries, and self-assessment questions. |
| (Strecher et al., 2008) | A randomized controlled trial examining the predictive value of engagement on 6-months abstinence rates, sociodemographic predictors of engagement, and what components predicted engagement in a tailored web-based intervention targeting smoking cessation. | 1866 smokers following a web-based intervention for smoking cessation in combination with nicotine replacement therapy were randomized into one of the 16 intervention arms. | Behavior change | Digital | ﻿The cumulative number of [intervention] sections opened by the participant. |
| (Suffoletto et al., 2021) | Pilot randomized control trial assessing the acceptability, engagement, and effects of a mobile intervention supporting mental health in young adults transitioning to college. | 52 young adults transitioning to college were recruited from primary care or a mental health clinic were randomized to receive the mobile support or enhanced usual care. | Mental health | Digital | Amount of text messaging and web-based responses. |
| (Young et al., 2021) | Comparison of engagement and non-usage attrition across 4 versions of a web-based dietary health promotion intervention from people experiencing symptoms of anxiety and low mood. | 614 online recruited participant were assigned to either of the 4 versions of the intervention. | Behavior change & mental health | Digital | 1) duration of program use in weeks, 2) total usage time, 3) total time key activities, 4) number of active sessions, 5) average time per session, 6) total number of completed key activities divided by total number of key activities, 7) number of goals set and marked as complete; and (8) percentage videos watched |
| **Questionnaire-based Measures** | | | | | |
| Author | Research aim | Sample and intervention type | Target context | Delivery mode | Operationalization of engagement |
| (Aizenstros et al., 2021) | Study aimed to investigate the degree of engagement and effectivity between an app-based behavior activation intervention for people with anxiety and depressive symptoms. | 208 participants who voluntarily downloaded the *MoodMission* app, and provided consent to participate. | Mental health | Digital | Homework rating scale |
| (Glenn et al., 2013) | Exploration of treatment dose and client engagement as predictors of treatment outcomes in a CBT intervention for anxiety disorders. | 503 patients with a panic disorder, generalized anxiety disorder, social anxiety disorder, and/or post-traumatic stress disorder chose to receive CBT, self-guided CBT, pharmacotherapy or a combination of pharmacotherapy and CBT. There was no pre-set intervention duration, since that was part of the measures of intervention engagement. | Mental health | In-person | ﻿1) Clinician rating of homework adherence, rated after every CBT session on a 4-point scale  2) ﻿Clinician rating of patient “overall commitment to CBT this session,” rated after every CBT session on a 0 to 10-point scale |
| (Graham et al., 2021) | Proof-of-concept evaluation of the association between subjective engagement with digital mental health interventions and changes in depression and anxiety outcomes. | 146 adults primary care patients with depression and anxiety symptoms were randomized to receive a digital mental health intervention or an 8-week waitlist, followed by treatment as usual. | Mental health | Digital | 19-item Usefulness, Satisfaction, and Ease of Use (USE) Questionnaire |
| (Kelders et al., 2020) | Evaluation of the psychometric properties of the TWentse Engagement with eHealth Technologies Scale (TWEETS) | 288 participants using a mobile step counting application of choice. | Behavior change | Digital | 1) behavioral engagement, 2) cognitive engagement, 3) affective engagement |
| (Lindner et al., 2014) | Study assessing the influence of different guidance media on the effectivity of and engagement with internet cognitive behavioral therapy for depression. | 38 wait-list clients suffering from major depressive disorder where recruited and randomized to receive a 7-week internet cognitive behavioral therapy intervention with 1) telephone guidance, or 2) email guidance. | Mental health | Digital | ﻿Self-rated estimates of how much time [participants] dedicated to their treatment each week and ratings of effort on a five-point scale corresponding to the range Very large to None at all (ranked 4–0). Therapists recorded the amount of time spent each week on each client. |
| (Mallonee et al., 2021) | Cross-sectional exploratory study assessing to what degree the clients’ perception of therapeutic alliance, therapist empathy, and coercion explain levels of engagement in outpatient clients with serious mental health conditions. | Anonymous online survey completed by 131 participants experiencing a serious mental health condition. | Mental health | In-person | Client version of the Engagement Measure |
| (McNealy & Lombardero, 2019) | Exploration of the correlations between mental health, treatment engagement and somatic symptoms in college students. | Cross-sectional survey of somatic symptoms, mental health and treatment engagement in 184 undergraduate college students. | Mental health | In-person | ﻿Yes/no to the questions “Have you ever received counseling or therapy for mental health concerns?” and: “Have you ever taken medication for mental health concerns?” |
| (Perski et al., 2019) | Validation of the Digital Behavior Change Interventions Engagement Scale (DBCI Engagement Scale) | 203 users of an app-based alcohol reduction program (i.e. *Drink Less*). | Behavior change | Digital | DBCI Engagement Scale assessing five elements of engagement:  1) amount of use, 2) depth of use, 3) attention, 4) interest, 3) enjoyment. |
| (Saul et al., 2016) | Investigation of disengagement and attrition in participants that disengaged after using an intervention once. | 132 participants that previously used an online intervention targeting smoking cessation, and did not return after initial use | Behavior change | Digital | Survey assessing the reasons for not returning to the [intervention] (four categories: life circumstances, [unhealthy behavior] related factors, [intervention] issues, and using other methods to [reduce unhealthy behavior]) |
| (Zelencich et al., 2019) | Investigation of the association between demographics, symptom severity, and therapy process factors and homework engagement in CBT for traumatic brain injury. | Analysis of 177 CBT sessions from 31 therapist-patient dyads of patients suffering from depressive and/or anxiety symptoms following traumatic brain injury. | Mental health | In-person | ﻿Observer version of the Homework Rating Scale (HRS-II) |
| **Qualitative Measures** | | | | | |
| Author | Research aim | Sample and intervention type | Target context | Delivery mode | Operationalization of engagement |
| (Fitzpatrick et al., 2017) | Assessment of feasibility and preliminary effectiveness of an automatic conversational agent in delivering CBT for depression and anxiety in young adults. | 70 young adults were randomized to either a 2-week period of CBT content delivered by an automatic conversational agent, of 2-week access to a self-help CBT eBook | Mental health | Digital | ﻿Qualitative assessment of participants thoughts on the best and worst thing about their experience [with the intervention] and to other comments |
| (Godlaski et al., 2009) | Qualitative study on the experiences and levels of engagement of rural woman entering substance-abuse treatment. | Semi-structured interviews with 12 rural woman that were in the beginning phase of an intensive outpatient treatment targeting substance abuse. | Mental health | In-person | Semi-structured interviews assessing the following themes:  - Circumstances leading towards treatment admission  - ﻿State of mind looking toward treatment entry  - ﻿Early experiences in treatment that made women feel more comfortable and why  - ﻿What made women feel uncomfortable and why  - ﻿Changes in the treatment program suggested by the women |
| (Knowles et al., 2015) | Qualitative exploration of patient experiences with computerized cognitive behavioral therapy for depression. | Semi-structured interviews with 36 patients suffering from depressive symptoms who attended a computerized cognitive behavioral intervention for depression. | Mental health | Digital | Semi-structured interviews, in which engagement was assessed using the following questions:  ﻿“How long did you use the program for? What helped you to use it/why did you stop using it?” |
| (Marker et al., 2019) | Assessment of the predicting role of motivation on treatment outcomes in a transdiagnostic cognitive behavioral group therapy for anxiety disorders. | Assessment of occurrence of change talk and counter change talk in 11 therapy groups (54 individual patients) and 12 sessions. | Mental health | In-person | Qualitative analysis of client language in group therapy that indicated steps taken towards (engagement) or away (disengagement) from change. |
| (Soderlund et al., 2021) | Qualitative analysis of interview data of a mixed-methods study on participants’ experiences with a transmedia storytelling intervention for Latina woman targeting mental health. | Qualitative analysis of previously collected interviews with 28 Latina woman with anxiety and/or depression symptoms who used the web-based intervention | Mental health | Digital | Semi-structured interviews using open-ended questions about the participants’ experience with the intervention, their perception of the avatar that was used in the intervention, and their attitudes about help-seeking. |
| **Mixed-methods Studies** | | | | | |
| Author | Research aim | Sample and intervention type | Target context | Delivery mode | Operationalization of engagement |
| (Gordon et al., 2021) | Mixed method study on the factors that influence engagement with a web-based self-help intervention for mental health and addiction complaints. | 542 participants recruited from 3 mental health facilities specialized in general psychiatry, substance use, crisis services, psychotherapy, post-traumatic stress disorder treatment, and treatment for borderline personality disorder were randomized to either access the intervention immediately, or after 3 months. | Mental health | Digital | Quantitative: Account activation, number of logins, and total time on the site.  Qualitative: repeated interviews assessing reasons for engagement and dis-engagement. |
| (Lawn et al., 2021) | Secondary analysis of a pre-existing dataset, assessing reasons for engaging in mental health services, reasons for dis-engaging with mental health services, and what services users re-engage with. | 535 service users or family members of service users who are engaged with Australian mental health services. | Mental health | In-person | Self-formulated questionnaire assessing use of service, reasons for service use, access, perceived quality, reasons for dis- and re-engagement, and qualitative questions about barriers and facilitators of engagement, and consequences of dis-engagement. |
| (Morrison et al., 2014) | Exploration of client engagement with internet delivered self-help interventions for non-clinical bowel problems due to poor life style | Mixed-method study assessing the experiences and engagement of 24 users of 3 intervention types: 1) general information, 2) self-assessment without tailored feedback, 3) self-assessment with tailored feedback. | Behavior change | Digital | Qualitative: participant experience with the intervention  Quantitative: Website Evaluation Questionnaire |
| (Newman et al., 2021) | Assessment of the effects of rurality on engagement in mental health treatment | Analysis of 998 medical files of patients from 24 mental health clinics in America. | Mental health | In-person | The number of counseling sessions documented in the medical record and self-reported medication use |
| (Yeager et al., 2018) | Study examining patient engagement with a digital trauma intervention | 440 patients with trauma symptoms that used a digital self-paced trauma intervention | Mental health | Digital | ﻿Subjective engagement: questions regarding frequency  and duration [of intervention use] on a 6-point scale ranging from 1 (“never”) to 6 (“more than once a day”). Duration was measured by the total estimated usage (in minutes) of the five modules.  Objective engagement: automatically recorded data of the frequency (number of pages visited) and duration {total number of minutes logged in) of intervention usage |
| **Review articles** | | | | | |
| Author | Research aim | Setting and intervention type | Target context | Delivery mode | Methodology |
| (Ng et al., 2019) | Review of measures and reports of user engagement indicators | Mobile app-based interventions for mental health | Mental health | Digital | Systematic review |
| (Short et al., 2018) | Overview of measurement options, considerations, and to provide directions for future research | eHealth and mHealth interventions for behavior change | Behavior change | Digital | Viewpoint |
| (Tetley et al., 2011) | Review of existing questionnaire-based measures for engagement, the constructs that are examined in these measures, the clinical utility and generalizability, and the psychometric properties | In-person psychotherapeutic interventions | Mental health | In-person | Systematic review |
